# Toxicity of six plant extracts and two pyridone alkaloids from *Ricinus communis* against the malaria vector *Anopheles gambiae*

**DOI:** 10.1186/1756-3305-7-312

**Published:** 2014-07-04

**Authors:** Sabina Wangui Wachira, Sabar Omar, Julia Wanjiru Jacob, Martin Wahome, Hans T Alborn, David R Spring, Daniel K Masiga, Baldwyn Torto

**Affiliations:** 1 International Centre of Insect Physiology and Ecology, P.O. Box 30772–00100, Nairobi, Kenya; 2 Kenya Medical Research Institute, Centre for Traditional Medicine and Drug Research, P. O. Box 54840–00200, Nairobi, Kenya; 3 Kenya Medical Research Institute, Centre for Biotechnology Research Development, P.O Box 54840–00200, Nairobi, Kenya; 4 Institute of Tropical Medicine and Infectious Diseases (ITROMID), Jomo Kenyatta University of Agriculture and Technology, P.O. Box 62000–00200, Nairobi, Kenya; 5 USDA/ARS-CMAVE, 1700 SW 23rd Dr., Gainesville, FL, USA; 6 Chemistry Department, University of Cambridge, Lensfield Road, Cambridge CB2 1EW, UK

**Keywords:** *Anopheles gambiae* s.s, Malaria, Mosquito, Alkaloid, Larvicidal, Toxicity, *Ricinus coummunis*, *Tithonia diversifolia*

## Abstract

**Background:**

The African malaria vector, *Anopheles gambiae* s.s., is known to feed selectively on certain plants for sugar sources. However, the adaptive significance of this behaviour especially on how the extracts of such plants impact on the fitness of this vector has not been explored. This study determined the toxicity and larvicidal activity on this vector of extracts from six selected plants found in Kenya and two compounds identified from *Ricinus communis*: 3-carbonitrile-4-methoxy-N-methyl-2-pyridone (ricinine), and its carboxylic acid derivative 3-carboxy-4-methoxy-N-methyl-2-pyridone, the latter compound being reported for the first time from this plant.

**Methods:**

Feeding assays tested for toxic effects of extracts from the plants *Artemisia afra* Jacq. ex Willd, *Bidens pilosa* L., *Parthenium hysterophorus* L.*, Ricinus coummunis* L., *Senna didymobotrya* Fresen. and *Tithonia diversifolia* Hemsl. on adult females and larvicidal activity was tested against third-instar larvae of *Anopheles gambiae* s.s. Mortality of larvae and adult females was monitored for three and eight days, respectively; Probit analysis was used to calculate LC_50_. Survival was analysed with Kaplan-Meier Model. LC-MS was used to identify the pure compounds.

**Results:**

Of the six plants screened, extracts from *T. diversifolia* and *R. communis* were the most toxic against adult female mosquitoes after 7 days of feeding, with LC_50_ of 1.52 and 2.56 mg/mL respectively. Larvicidal activity of all the extracts increased with the exposure time with the highest mortality recorded for the extract from *R. communis* after 72 h of exposure (LC_50_ 0.18 mg/mL). Mosquitoes fed on solutions of the pure compounds, 3-carboxy-4-methoxy-N-methyl-2-pyridone and ricinine survived almost as long as those fed on the *R. communis* extract with mean survival of 4.93 ± 0.07, 4.85 ± 0.07 and 4.50 ± 0.05 days respectively.

**Conclusions:**

Overall, these findings demonstrate that extracts from the six plant species exhibit varying bioactivity against the larvae and adult females of *An. gambiae* s.s. *T. diversifolia* and *R. communis* showed highest bioactivity against adult females *An. gambiae* and larvae while longevity of female *An. gambiae* s.s. decreased with exposure time to the two pure compounds.

## Background

Malaria remains a life-threatening disease caused by parasites that are transmitted to humans through the bites of certain species of infected anopheline mosquitoes [1]. The WHO global estimates document about 207 million cases of malaria and an estimated 627,000 deaths in 2012 [1]. Most malaria-related deaths occur in children under 5 years of age in sub-Saharan Africa [1]. The high mortality burden persists under the backdrop of rising resistance to standard anti-malarials especially by *Plasmodium falciparum*, the most widespread etiological agent for human malaria. In fact, currently, malaria treatment is threatened by resistance to all anti-malaria drugs including artemisinin-based combination therapies [2-5], the first line treatment for *P. falciparum* malaria. Therefore, continued efforts to develop alternative therapies are necessary.

The principal vector of *P. falciparum* in sub-Saharan Africa is *An. gambiae* Giles *s.s.*[6,7]. Knowledge of the vector’s interaction with plants can lead to the development of new malaria control tools. Plant feeding influences important aspects in the life cycle of malaria vectors such as their survival and reproductive success. After emergence, both males and females almost exclusively feed on plant-derived sugary fluids including sap, nectar, and honeydew in order to supplement their energy reserves and to sustain life [8,9].

However, plant feeding by *Anopheles gambiae s.s*. on host plants is known to occur in a discriminative manner [10-12]. It is commonly known that host plants are sought for sugar and from an evolutionary perspective, those with high sugar content are likely to be more attractive than their counterparts with lower sugar content. However, the association between the sugar content and plant attractiveness to this mosquito vector remains equivocal. Manda *et al.,*[11] showed that there was correlation in the sugar content and the degree of attractiveness of *An. gambiae* to preferred plant species: *Tecoma stans*, *S. didymobotrya*, *R. communis* and *Hamelia patens*, but not *P. hysterophorus*. Nonetheless, a related study showed that the attractiveness of *An. gambiae* to *P. hysterophorus* was not only related to volatile compounds produced by this plant but to higher sugar levels [10,13]. Regardless of plant sugar content, these findings suggest that plant feeding may play other important roles in the life of this mosquito vector.

To expand on an earlier study which reported the attraction of *An. gambiae* to three host plants; *P. hysterophorus, B. pilosa* and *R. coummunis*[10], we have evaluated the extracts of these plants for their toxicity and larvicidal activity. We also included three plants (*T. diversifolia, S. didymobotrya, A. afra*) of ethnobotanical and medicinal values commonly used in the treatment of malaria in Kenya and two compounds isolated from R*. communis*. Additionally, *Azadiractin indica* A. Juss. was included as a positive control because of its known broad spectrum bioactivity against a wide range of insects.

## Methods

### Plant materials

*P. hysterophorus* (Asteraceae, Voucher No. SW00010), *T. diversifolia* (Asteraceae, Voucher No. SW00015), *B. pilosa* (Asteraceae, Voucher No. SW00005), and *R. communis* (Euphorbiaceae, Voucher No. SW00013) were all collected from Nairobi county (1°13′23.46″ S, 36°53′46.83″ E), Kenya in April 2011. *S. didymobotrya* was collected from Laikipia County (0°01′39.43″ N 37°03′38.23″ E), Kenya (Leguminosae, Voucher No. SW00018) and *A. afra* (Asteraceae, Voucher No. SW00021) from Meru county (0°09′17.75″ N 37°39′37.23″ E), Kenya in July 2012. *A. indica* (neem) (Meliaceae, Voucher No. SW00023) was collected from Kwale county (4°16′45.83″ S 39°35′37.38″ E), Kenya in December 2012. The plants were authenticated by a botanist and voucher specimen preserved at the National Museums of Kenya, Nairobi. *A. indica* was selected as a positive control because it has broad spectrum bioactivity against a wide range of insects and parasites including *An. gambiae*[14-16].

### Extraction

The fresh leaves (250 g each) of seven plants species listed in Table 1 were crushed in a mortar with a pestle to form a paste. The paste was then extracted with 750 mL of methanol (Analytical grade, Fluka) for 72 h, and then filtered. The procedure was repeated three times and the filtrate evaporated to dryness under vacuum. The concentrated extracts were stored at −20°C until needed for bioassays.

**Table 1:** List of selected plant species

**Botanical name**	**Voucher specimen number**	**Common name**	**Family**
*Artemisia afra* Jacq. ex Willd.	SW00021	Wormwood	Asteraceae
*Bidens pilosa* L.	SW00005	Black jack	Asteraceae
*Parthenium hysterophorus* L	SW00010	Wild quinine	Asteraceae
*Tithonia diversifolia* Hemsl.	SW00015	Mexico sunflower	Asteraceae
*Ricinus communis* L.	SW00013	Castor oil bean	Euphorbiaceae
*Senna didymobotrya* Fresen	SW00018	African senna	Leguminosae
*Azadirachta indica* A. Juss. (neem)	SW00023	Mwarubaini	Meliaceae

### Isolation and identification of compounds from *R. communis*

*R. communis* gave the highest bioactivity against both adult females and larvae of *An. gambiae* s.s, therefore, compounds were isolated from this plant. The methanol extract of *R. communis* was subjected to vacuum liquid chromatography using silica gel (0.032-0.062 mm, Sigma aldrich) eluting with hexane, ethyl acetate, methanol and water. Three fractions were obtained. Fraction 2 was subjected to repeated column chromatography eluting with ethyl acetate, methanol and water and this yielded two pure compounds identified by LC-MS as ricinine and 3-carboxy-4-methoxy-N-methyl-2-pyridone. The LC-MS analysis was carried out on an Agilent 1100 HPLC system (Agilent Technologies, USA), equipped with a Spectra System Pump P4000, an on-line degasser, an auto-sampler AS3000, a column oven temperature of 60°C, and an ultraviolet detector UV6000LP coupled with a XCalibur software version 2.0 SUR1 (Agilent Technologies, USA). The separation was carried out on a Varian PLRP-S 100 Å polymer column (4.6 mm × 250 mm, Agilent Technologies, USA) and temperature was set at 60°C. The mobile phase consisted of A: H_2_O 0.1% Formic acid, B: 90% CH_3_CN/H_2_O 0.1% formic acid at a flow rate of 1 ml/min, split 9:1 after UV detector to give a flow of 100 ul/min to the LC/MS, using the following gradient elution: 90% A for 2 min, linear gradient to 95% B at 15 min and at 95% B for 10 min followed by 5 min return to 90% A. The injection volume was 20 μl.

The analysis was performed on a Finnigan LCQ Deca XP Maxi, ion trap mass spectrometer (Thermo Finnigan, USA) connected to the Agilent 1100 HPLC instrument via an electrospray ionization (ESI) interface. The chromatographic conditions were the same as described above. The MS spectra were acquired in both negative and positive modes and full scan mass spectra were acquired from the mass-to-charge ratio (*m*/*z*) of 100–800. Parameters for MS were as follows: Aux sweep gas 5 au, ion spray voltage 5 kV; capillary temperature 280°C.

### Insects

Mosquitoes used for the experiments were obtained from established laboratory-reared colonies of *An. gambiae s.s.* (Mbita strain) at the International Centre of Insect Physiology and Ecology (ICIPE) Duduville campus, Nairobi, Kenya. The strain was initially collected as larvae from anopheline pools at Mbita Point, Suba District, Nyanza County, Western Kenya in April 2011. Larvae were reared in plastic trays (39 × 28 × 14 cm deep) in an insectary at a density of about 500 larvae per 3 L of distilled water. The rearing room was maintained at 32 ± 2°C, and 52% relative humidity (R.H.). The larvae were fed daily on (3 mg/larvae/day) Tetramin® fish food (Tetra, Germany). The adult mosquitoes were kept in cubic cages (30 × 30 × 30 cm) in a separate room maintained at 26 ± 2°C, 70–80% R.H. with a photoperiod of LD 12:12 h, the light being provided by a fluorescent lamp (40 watt). Both male and female mosquitoes were kept together after emerging and were separated during the assay. Mosquitoes were fed on 6% glucose solution *ad libitum* after emergence. The conditions in the bioassay rooms were the same as those of the rearing room.

### Toxicity test

Toxicity tests were carried out using feeding assays. Briefly, two groups of female *An. gambiae s.s.* (3–5 days old) and previously starved for 12 h were released into the experimental cages (15 × 15 × 15 cm) (200/cage) and left to acclimatize for 1 h. One group was fed on the plant extract dissolved in 0.1 mL of dimethyl sulfoxide (DMSO) and 19.9 mL of 6% sugar solution contained in a 20 mL vial, while the control group fed on a similar extract-free solution. The test extract and control were then introduced into the centre of their respective cages and the mosquitoes fed on the test and control solutions through an immersed rolled up filter paper (Whatman No. 1) with 5 cm of it exposed above the top of the 20 mL vial. For the plant extract, five different concentrations; 1, 3, 5, 7 and 10 mg/mL of the extract were tested in three replicates per dose. Daily mortality was recorded in all the mosquito groups for eight consecutive days.

### Larvicidal activity

Larvicidal activity was carried out as described by the WHO [17] with minor modifications as described by Rahuman *et al*., [18] using third instar larvae. Four different concentrations; 0.125, 0.25, 0.5 and 1 mg/mL were tested. The extracts were prepared in 0.1 mL of DMSO and 99.9 mL of distilled water contained in a 250 mL beaker. This solvent system in a similar ratio served as the control. Three batches of twenty larvae were used for all the experiments and the number of dead larvae in each test was counted and removed after 24, 48 and 72 h of exposure. The mortality data were subjected to Probit analysis to calculate lethal concentration values (LC_50_ and LC_90_) and lower and upper 95% fiducial limits. LC50, LC90 and Chi-square values were calculated using the EPA (U.S. Environmental Protection Agency) computer Probit analysis program (version 1.5).

### Survival assay

Survival tests for the pure isolates from *R. communis* was carried out using the feeding assay described above (see Toxicity test). We aimed to test how the two isolates compared with the crude extract, and in preliminary dose response assays, we found that ricinine at 0.04 mg/ml was toxic to *An. gambiae s.s.* adult females. Therefore, we compared this relatively high dose of ricinine and its derivative 3-carboxy-4-methoxy-N-methyl-2-pyridone to that of the crude extract presented to adult female mosquitoes also at this dose. Additionally, two groups of mosquitoes had access to distilled water and 6% glucose representing negative and positive controls, respectively. Tests were carried out in five replicates. SPSS Advanced Statistics 20.0 was used for data management. Survival was analysed with Kaplan-Meier Model. The mean and median survival time are reported with their 95% confidence interval (CI). The median survival time is the time at which half of the female *Anopheles gambiae* died. If the survival curve does not fall to 0.5 (50%) then the median time cannot be computed as in the case of glucose.

## Results

### Toxicity test

Table 2 shows the results of the toxic effects of the different plant extracts against adult females of *An. gambiae s.s* compared to that of the positive control *A. indica*. Toxicity varied with the plant extract and adult female feeding time. After 3 days of mosquito feeding, the highest mortality was recorded for extracts from *T. diversifolia* and *R. communis* with LC_50_ values of 8.30 and, 8.69 mg/mL, respectively, which compared to that of the positive control *A indica* (LC_50_ 8.69 mg/mL). *P. hysterophorus, A. afra, B. pilosa* and *S. didymobotrya* were less toxic with LC_50_ values of 18.90, 19.56, 21.09 and 23.39 mg/mL respectively. Toxicity of the different extracts increased with increasing mosquito feeding time, but the pattern of toxicity observed across the different plant extracts after 7 days remained virtually the same with *T. diversifolia* and *R. communis* recording the highest mortality with LC_50_ values of 1.53 and, 2.56 mg/mL, respectively. *T. diversifolia* compared well with *A. indica* (LC_50_ 1.34 mg/mL). *A. afra, B. pilosa, P. hysterophorus* and *S. didymobotrya* were less toxic with LC_50_ values of 4.16, 4.79, 5.74 and 8.21 mg/mL respectively (Table 3).

**Table 2:** **Relative toxicity of different plant extracts against female *An. gambiae s.s. *** **after 3 days of oral feeding**

**Plant extract**	**χ**^**2**^	**Regression equation**	**LC**_**50 **_**(mg/mL)**	**LCL-UCL(mg/mL)**	**LC**_**90 **_**(mg/mL)**	**LCL-UCL(mg/mL)**
*A. afra*	1.99	y = 3.38 + 1.26x	19.56	15.93 - 25.84	204.48	119.95 - 434.91
*B. pilosa*	0.97	y = 2.94 + 1.56x	21.09	17.31 - 27.70	140.35	87.87 - 272.85
*P. hysterophorus*	1.76	y = 2.35 + 2.08x	18.90	15.61 - 25.44	78.28	49.85 - 161.91
*T. diversifolia*	2.17	y = 3.71 + 1.40x	8.30	7.53 - 9.28	67.86	50.99 - 97.14
*R. communis*	3.61	y = 2.65 + 2.14x	8.69	11.28 - 14.25	49.53	37.89 - 70.97
*S. didymobotrya*	2.33	y = 1.84 + 2.30x	23.39	18.29 - 35.67	84.27	50.33 - 208.19
*A. indica*	1.37	y = 3.58 + 1.51x	8.69	7.86 - 9.75	61.17	45.68 - 89.40

LC_50_ and LC_90_ lethal concentration that kills 50% and 90% of the ingesting female *An. gambiae s.s.* respectively, LCL lower confidence limit, UCL upper confidence limit based on 95% Confidence interval, χ^2^ chi-square values.

**Table 3:** **Relative toxicity of different plant extracts against female *An. gambiae s.s. *** **after 7 days of oral feeding**

**Plant extract**	**χ**^**2**^	**Regression equation**	**LC**_**50 **_**(mg/mL)**	**LCL-UCL(mg/mL)**	**LC**_**90 **_**(mg/mL)**	**LCL-UCL(mg/mL)**
*A. afra*	7.44	y = 4.14 + 1.38x	4.16	3.76 - 4.58	34.91	27.85 - 46.24
*B. pilosa*	66.18	y = 2.27 + 4.01x	4.79	2.11 - 6.52	9.99	7.18 - 45.66
*P. hysterophorus*	28.64	y = 2.48 + 3.31x	5.74	3.76 - 7.45	13.99	9.89 - 45.50
*T. diversifolia*	11.76	y = 4.55 + 2.39x	1.53	1.07 - 1.96	5.23	4.19 - 7.00
*R. communis*	36.02	y = 3.47 + 3.73x	2.56	1.41 - 3.40	5.65	4.36 - 8.66
*S. didymobotrya*	30.57	y = 1.70 + 3.60x	8.21	6.07 - 13.38	18.61	12.12 - 222.43
*A. indica*	30.04	Y = 4.69 + 2.50x	1.34	0.67 - 1.94	4.34	3.08 - 7.29

LC_50_ and LC_90_ lethal concentration that kills 50% and 90% of the ingesting female *An. gambiae s.s.* respectively, LCL lower confidence limit, UCL upper confidence limit based on 95% Confidence interval, χ^2^ chi-square values.

### Larvicidal activity

Tables 4 and 5 summarize the dose-dependent larvicidal activity of the plant extracts against third-instar larvae of *An. gambiae s.s.* after 24 h and 72 h, respectively. The mortality of larvae varied with the plant extract and larval exposure time. After 24 h of larval exposure, the highest mortality was recorded for extracts from *R. communis* with LC_50_ values of 0.40 mg/mL, which was in the same range as the positive control *A indica* (LC_50_ 0.44 mg/mL). *S. didymobotrya, B. pilosa, T. diversifolia, A. afra* and *P. hysterophorus* caused low mortality with LC_50_ values ranging between 0.53 and 1.88 mg/mL respectively. The larvicidal activity of the different extracts increased with increasing larval exposure time, however, the pattern of activity observed across the different plant extracts after 72 h remained the same with *R. communis* recording the highest mortality with LC_50_ values of 0.18 mg/mL followed closely by *B.* pilosa, *A. afra* and *P. hysterophorus* all the three with LC_50_ values of 0.21 mg/mL which were on the same range with positive control *A indica* (LC_50_ 0.19 mg/mL). *S. didymobotrya* and *T. diversifolia* caused the lowest mortality with LC_50_ values of 0.27, and 0.33 mg/mL respectively.

**Table 4:** **Larvicidal activity of plant extracts against 3**^**rd **^**Instar larvae of *An. gambiae s.s. *** **after 24 h of exposure**

**Plant extract**	**χ**^**2**^	**Regression equation**	**LC**_**50 **_**(mg/mL)**	**LCL-UCL(mg/mL)**	**LC**_**90 **_**(mg/mL)**	**LCL-UCL(mg/mL)**
*A. afra*	3.03	y = 5.03 + 2.07x	0.96	0.75 - 1.44	4.01	2.34 - 10.83
*B. pilosa*	0.81	y = 5.26 + 1.25x	0.62	0.45 - 1.02	6.54	2.77 - 47.25
*P. hysterophorus*	3.52	y = 4.61 + 1.41x	1.88	1.13 - 6.17	15.21	5.04 - 250.69
*T. diversifolia*	2.73	y = 5.35 + 2.63x	0.74	0.62 - 0.94	2.27	1.60 - 4.04
*R. communis*	0.79	y = 6.13 + 2.87x	0.40	0.35 - 0.47	1.13	0.89 - 1.59
*S. didymobotrya*	0.52	y = 5.86 + 3.08x	0.53	0.46 - 0.62	1.37	1.08 - 1.97
*A. indica*	0.67	y = 5.899 + 2.54x	0.44	0.38 - 0.53	1.41	1.06 - 2.18

LC_50_ and LC_90_ lethal concentration that kills 50% and 90% of *An. gambiae s.s.* larvae exposed to the extracts, LCL lower confidence limit, UCL upper confidence limit based on 95% Confidence interval, χ^2^ chi-square values.

**Table 5:** **Larvicidal activity of plant extracts against 3**^**rd **^**Instar larvae of *An. gambiae s.s. *** **after 72 h of exposure**

**Plant extract**	**χ**^**2**^	**Regression equation**	**LC**_**50 **_**(mg/mL)**	**LCL-UCL(mg/mL)**	**LC**_**90 **_**(mg/mL)**	**LCL-UCL(mg/mL)**
*A. afra*	3.074	y = 6.98 + 2.94x	0.21	0.18 - 0.25	0.58	0.47 - 0.78
*B. pilosa*	1.75	y = 7.10 + 3.08x	0.21	0.18 - 0.24	0.54	0.45 - 0.72
*P. hysterophorus*	0.15	y = 6.44 + 2.09x	0.21	0.16 - 0.25	0.84	0.63 - 1.33
*T. diversifolia*	3.49	y = 6.94 + 4.00x	0.33	0.29 - 0.37	0.68	0.58 - 0.86
*R. communis*	0.91	y = 8.17 + 4.21x	0.18	0.15 - 0.20	0.36	0.30 - 0.45
*S. didymobotrya*	2.98	y = 6.93 + 3.36x	0.27	0.23 - 0.30	0.64	0.53 - 0.83
*A. indica*	2.758	y = 7.00 + 2.73x	0.19	0.15 - 0.22	0.55	0.44 - 0.75

LC_50_ and LC_90_ lethal concentration that kills 50% and 90% of *An. gambiae s.s.* larvae exposed to the extracts, LCL lower confidence limit, UCL upper confidence limit based on 95% Confidence interval, χ^2^ chi-square values.

### Survival tests

**Figure 1:**
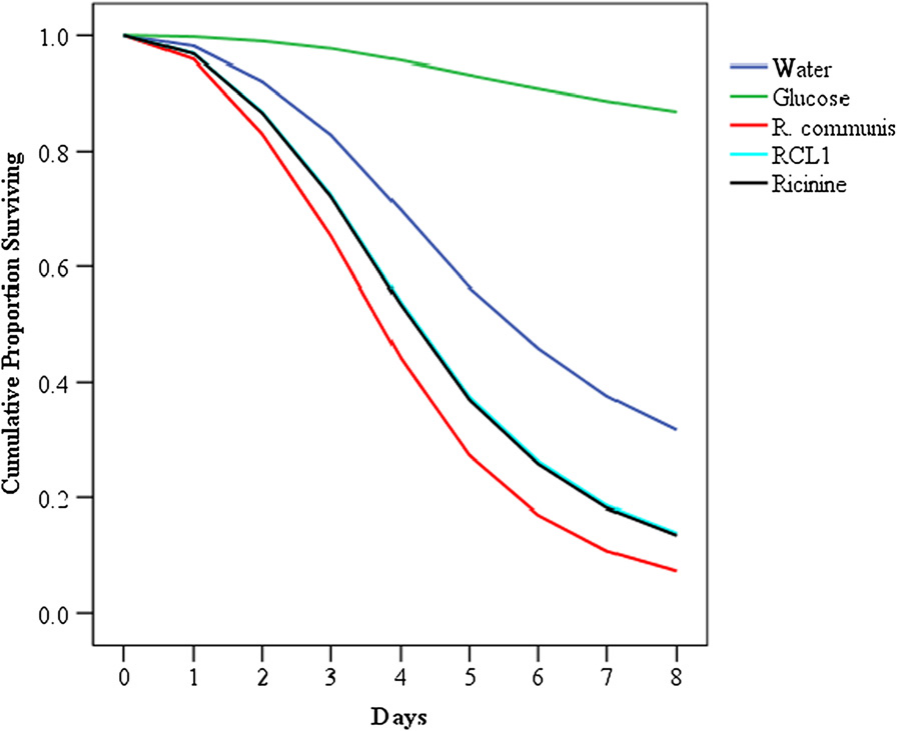
**Survival curves of female *An. gambiae s.s. *** **on exposure to *R. communis *** **extract, 3-carbonitirle −4-methoxy-N-methyl-2-pyridone (ricinine), 3-carboxy-4-methoxy-N-methyl-2-pyridone (RCL1), distilled water and 6% glucose.**

Figure 1 shows survival curves of female *An. gambiae* exposed to *R. communis* extract, 3-carboxy-4-methoxy-N-methyl-2-pyridone, ricinine, distilled water and 6% glucose. The glucose-fed group had the longest overall survival, with mean survival time of 7.65 ± 0.04 days (Figure 1, Table 6). The water-fed group gave the next longest survival time of 5.79 ± 0.07 days. Mosquitoes fed on 3-carboxy-4-methoxy-N-methyl-2-pyridone and ricinine survived almost as long as those fed on the *R. communis* extract with mean survival of 4.93 ± 0.07, 4.85 ± 0.07 and 4.50 ± 0.05 days respectively (Table 6).

**Table 6:** **Mean and median survival times of female *Anopheles gambiae *** **maintained on 0.04 mg/ml of different samples**

**Diet**	**Mean ± SE (days)**	**95% CI LCL-UCL**	**Median ± SE (days)**	**95% CI LCL-UCL**
Water	5.79 ± 0.07	5.65-5.92	6.00 ± .014	5.72-6.28
Glucose	7.65 ± 0.04	7.58-7.73	.	.
*R. communis*	4.50 ± 0.05	4.40-4.60	5.00 ± 0.06	4.89-5.11
RCL1	4.93 ± 0.07	4.80-5.06	5.00 ± 0.10	4.81-5.19
Ricinine	4.85 ± 0.07	4.71-4.99	4.00 ± 0.11	3.78-4.22

SE, standard error; CI, 95% confidence interval, LCL- lower confidence limit, UCL- upper confidence limit, RCL1- 3-carboxy-4-methoxy-N-methyl-2-pyridone.

### Identification of compounds

Ricinine: this compound was obtained as white crystals. The mass spectrum showed the M + 1 peak (base peak) at m/z 165 (C_8_H_8_N_2_O_2_^+^). The other prominent peaks were those at m/z 149(C_7_H_5_N_2_O_2_^+^), 138 (C_7_H_8_NO_2_^+^) and 109 (C_6_H_7_NO^+^). This was compared with the literature and confirmed that the compound was ricinine, having similar fragments.

**Figure 2:**
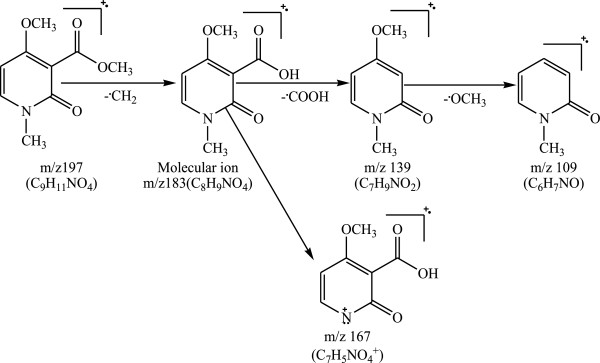
Fragmentation pattern for 3-carboxy-4-methoxy-N-methyl-2-pyridone.

3-carboxy-4-methoxy-N-methyl-2-pyridone: this compound is reported for the first time in *R. communis*. It was obtained as white powder which dissolved in methanol and sparingly in acetone. The mass spectrum showed the M + CH_2_ peak at m/z 197 (C_9_H_11_NO_4_^+^). The molecular ion peak was observed at m/z 183 (C_8_H_9_NO_4_^+^). Other prominent peaks were those at m/z 166 (base peak) (C_8_H_8_N_2_O_3_^+^), 139 (C_7_H_9_NO_2_^+^) and 109 (C_6_H_7_NO^+^). Figure 2 provides the possible fragmentation pattern for this molecule to justify some of the observed peaks in the mass spectrum.

## Discussion

Plant extracts may exhibit varying bioactivity on insects. This study showed that of the six extracts evaluated, those from *T. diversifolia,* and *R. communis* caused the highest mortality in females of *An. gambiae.* Previous studies have reported the presence of sesquiterpene lactones in *T. diversifolia* and alkaloids in *R. communis*[19,20]. These two classes of compounds are known to exhibit a wide range of biological activity including against insect vectors [21,22], which may explain the observed effect of these two plants. Indeed, the toxic effects of extracts from these two plants were comparable to that of the positive control, *A. indica*, which is known to contain limonoids, well known as anti-insect compounds and found to be effective against the mosquitoes *Aedes aegypti* and *An. gambiae*[23-26]. The differential mortality effect observed for the plants evaluated especially among those known to be attractive to this vector justifies the fact that plant feeds may have additional effects on this mosquito and are important not only for sugars. Therefore, the higher activity found for extracts from *T. diversifolia* and *R. communis* relative to the other related Asteraceae plants and for the other families could be due to differences in the secondary metabolites present in the different plants.

We observed a relatively high larvicidal activity of the extract from *R. communis* compared to the extracts from the other five plants, a finding which is in agreement with a previous study, which showed effectiveness of the extract from this plant with potent mosquito larvicidal acitivity against larvae of *Anopheles arabiensis*[27]. In contrast, we observed a rather weak larvicidal activity of *T. diversifolia* extracts. Although the reason for this observation is unclear, it could be related to the low concentration of the extract from this plant tested in this study as a previous study showed high activity against *Aedes aegypti* at higher doses > 50 mg/mL tested [28].

The influence of exposure time on mosquito survival was linked to the amount of test material consumed, which varied depending upon the sample. The group of mosquitoes feeding on glucose, as expected survived the longest period followed by those fed on water. Interestingly, our results show that the pattern of survival on the extract from *R. communis* and the two pure isolates from this plant was similar. They all lowered the survival of mosquitoes, which decreased with time compared to those fed on glucose and water only. The survival of mosquitoes on the two isolates from *R. communis* was superimposable on one another suggesting that substitution at the 3-position with a carbonitrile or carboxylic acid did not affect the activity of the alkaloid. Thus activity of the alkaloid may involve other moieties or the combined effect of several of these spread across the molecule. It is worthy to note that the crude extract was slightly more active than the individual isolates suggesting that other minor components in the extract could play a role in the overall activity of the extract.

## Conclusion

We conclude that, of the six plants, extracts from *T. diversifolia* and *R. communis* could serve as useful candidate insecticides against larvae and adult females of *An. gambiae* s.s. since they showed the highest bioactivity.

## Competing interests

The authors declare that they have no competing interests.

## Authors’ contributions

SWW & BT conceived and designed the study. SWW, JW, MW performed the experiments and SWW analyzed the data. SWW, SO, DM, DS, AH, BT wrote the paper. All authors approved the final version for submission.
